# Immune reconstitution inflammatory syndrome in a unique clinical scenario

**DOI:** 10.4103/2589-0557.75031

**Published:** 2010

**Authors:** G. Anubhav, N. K. Kamath

**Affiliations:** Department of Dermatology, Venereology and Leprosy, RNT Medical College, Udaipur, Rajasthan, India; 1 Department of Dermatology, Venereology and Leprosy, Kasturba Medical College, Mangalore, Karnataka, India

**Figure 1:**
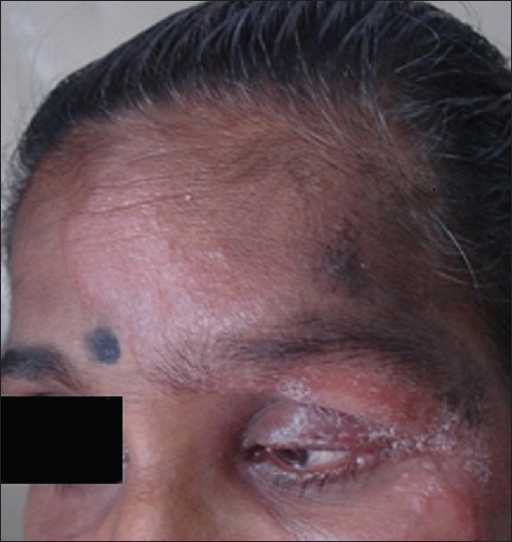
Infiltrated, erythematous plaque, extending from the forehead to eyelids with a satellite lesion

Sir,

A 37-year-old female patient on antiretroviral therapy (ART; Stamivudine, Lamivudine, Efavirenz) for 2.5 months, presented with the complaints of single, reddish, raised lesion on the forehead of 15 days’ duration associated with pain and watering from the eyes. There was no history suggestive of Hansen’s disease. Her husband had died 2 years ago of HIV disease. On clinical examination, the patient was found to have jaundice with generalized lymphadenopathy. Systemic examination was within normal limits. Cutaneous examination showed single, tender, erythematous, infiltrated plaque (10 × 8), irregular in shape with a satellite lesion, over the forehead, extending to the both eyelids. [Figure 1]. Sensations were intact and the left supraorbital nerve was thickened and tender. Motor examination revealed no abnormality. The patient had also been taking anti-tubercular treatment (ATT) since last 1.5 months. At the commencement of ART, her CD4 count was 67 cell/mm^3^, which rose to 258 cells/mm^3^ after 2.5 months of ART. The patient had anemia (Hb 7.2 g%). ESR was 120 mm of Hg. Liver function tests and renal function tests were normal. Biopsy from the plaque showed numerous ill-defined, noncaseating tuberculoid granulomata made up of epitheloid cells, Langerhan’s giant cells, and lymphocytes, associated with dermal edema. A diagnosis of borderline tuberculoid (BT) Hansen’s disease in type 1 reaction as an immune reconstitution inflammatory syndrome (IRIS) was considered. The patient was administered systemic steroids with PB-MDT, with continuation of ART and ATT.

The introduction of ART has led to emergence of a new clinical syndrome, IRIS. IRIS can be defined as a pathologic inflammatory response to pre-existing microbial, host or other antigens that result in paradoxical deterioration in an HIV-infected person after initiating ART.[1] This immune response can be either against an infectious agent or a noninfectious condition, the former being more common than the latter.[1,2] The suppression of HIV replication allowing gradual restoration of immunity is central to the pathogenesis of IRIS. Shelburne *et al*.[3] had proposed the following set of criteria for the diagnosis of IRIS:

A patient must

- be HIV positive and receiving ART;
- have a decreasing viral load, with or without an increase in the CD4 cell count from baseline;
- have clinical symptoms consistent with an inflammatory process in which the clinical course is not consistent with the expected course of previously diagnosed opportunistic infections (OIs), expected course of newly diagnosed OIs, or drug toxicity.

IRIS occurs most commonly when the CD4 cell count is less than 200 cells/mm^3^. It tends to occur 1–6 months after initiation of ART and represents the restoration of immunity that is immunopathologic rather than protective.[4] IRIS in leprosy is characterized by the development of type 1 reactional state in an unstable form of leprosy. [5] Lysis of bacilli takes place due to improved immunity following ART causing an inflammatory reaction, presenting as IRIS. The clinical course and therapeutic response of *Mycobacterium leprae* and HIV co-infection has been the subject of debate. Nery *et al*. concluded that an HIV co-infection did not seem to change the natural course of the disease, or cause difficulties in diagnosis and treatment.[6] The co-infection of HIV and leprosy is a rare event in endemic areas for leprosy and HIV, such as India. There is no change in the clinical spectrum of leprosy, leprosy reactions, and neuritis among co-infected patients.[5] Histopathological observations reveal a normal spectrum of appearance in biopsy of leprosy lesions from co-infected patients suggesting that a cell-mediated immune response to *M. leprae* is preserved at the site of the disease.[5] HIV patients concomitantly infected with leprosy may not show any signs of leprosy, due to diminished cell-mediated immunity (CMI), but could well be the source of infection in a healthy community.[3] All co-infected patients respond to the regular treatment but chances of relapse are more. Therefore, a longer duration of surveillance is advisable after fixed duration therapy.[5]
